# The Glasgow Western Infirmary

**Published:** 1897-11-13

**Authors:** 


### The Hospital

A Journal of

The Medical Sciences and Hospital Administration.

Vol. XXIII.—No. 581.] [Nov. 13, 1897

### The Glasgow Western Infirmary.

The unfortunate differences which have arisen in the Glasgow Western Infirmary, in consequence of the decision of the managers to appoint an additional surgeon, and, in order to provide him with material, to deprive Professor Macewen of a ward containing eighteen beds, are matters which can no longer be described as possessing merely a local interest, inasmuch as they not only affect the education of students intending to graduate in the University of Glasgow, but have also served to raise the very important question of whether the surgeon in charge of a case, or some other, and possibly non-medical authority, should be the proper person to judge of the character and amount of the nursing required by the patient. Professor Macewen, of whose eminence as a surgeon and teacher it is unnecessary to speak, complains that the loss of the ward of which the managers have deprived him leaves him with only one male and one female ward, in each of which, therefore, both accidents and chronic cases must be received, often to the detriment of the latter from the noise and bustle inseparable from the admission and treatment of the former. He complains, still more strongly, that the diminished number, and hence the diminished variety, of the chronic cases left to him seriously curtails his material for teaching, and exposes the patients who remain to a larger amount of examination from students than they would otherwise be called upon to undergo. Apart from this consideration, it is manifest that a diminished number of cases in a clinical ward may constitute a very grave evil as an impediment to instruction. A clinical teacher can scarcely have too much material, if not to use, at least to select from, because he must often desire opportunities of illustrating points of contrast between conditions which are liable to be mistaken for one another. The intentions of the managers have in all probability been excellent, but, in the words of Faraday, they appear to have arrived at a conclusion without due knowledge of the premises. According to our latest advices, the Senate of the University has officially declared that the withdrawal of the ward leaves the professor of clinical surgery without the "reasonable provision" which the Senate is bound to require; and we presume, therefore, that the managers will be compelled to abandon the position which they have somewhat rashly occupied. We trust that this portion of the dispute may be settled by the construction of a convenient bridge for the departure of the enemy.

On the other question on which differences have arisen between the Professor and the managers, it is impossible for anyone conversant with the needs of patients to entertain a doubt. Professor Macewen had his ward largely occupied by very urgent cases; and he complained, first, that the hours of duty were so arranged that, on his visit in the morning he could not see the nurses who had been in charge during the night; and, secondly, that, even in emergencies, and although he offered to defray the increased cost out of his own pocket, he could not obtain nurses in sufficient number for the work that had to be done. At one time, there were in his three wards thirty-one patients who were entirely helpless, several of them having recently undergone very grave operations, and all of them requiring special and almost constant attendance. To meet these requirements, there were three nurses in each ward, with a sister in charge of the three wards; and, of the nine nurses, only one had been fully trained, the others being apparently learners or probationers. Professor Macewen applied to the superintendent of the infirmary for additional help, and it was refused; although, at the very same time, an extra nurse was brought from outside in order to meet a similar demand from another member of the staff. In the opinion of the managers, presumably, the superintendent, who does not appear to be a medical man, was better qualified than Professor Macewen to decide upon the amount of nursing required by patients under the Professor's own care; and it seems hardly necessary to say that such a contention appears to afford absolute proof of the utter unfitness of these managers for the proper discharge of the responsibilities which they have undertaken. The absurdity of the position which they appear to have assumed is such that it cannot be made more manifest by any addition to the plainest statement of the facts. We presume, as a matter of high probability, that the present stage of the controversy can only have been reached after a period of prolonged friction, by which personal or party feelings of great acuteness may have been aroused, insomuch that the managers, and perhaps also the superintendent, may have been more solicitous to thwart and annoy the Professor than to provide for the welfare of the institution committed to their charge. If this be so, it will be well for them to remember that competent managers and superintendents are as plenty as blackberries; but that there is only one Professor Macewen in her Majesty's dominions.

